# 2-Octyl Cyanoacrylate (Dermabond®) Inhibits Bridging Bone Formation of Articular Fractures in a Rat Model

**DOI:** 10.7759/cureus.16758

**Published:** 2021-07-30

**Authors:** Vincent Gomez, Mark Cairns, Paul Weinhold, Alexander D Jeffs, Benjamin Bortner, Anthony V Paterno, Laurence Dahners, Reid W Draeger

**Affiliations:** 1 Department of Orthopaedic Surgery, University of North Carolina at Chapel Hill, Chapel Hill, USA

**Keywords:** intraarticular fracture, 2-octyl-cyanoacrylate, bone bridging, rat model, orif

## Abstract

One technique often used for small intraarticular fracture fixation involves the use of 2-octyl-cyanoacrylate (2-OCTA) (Dermabond®, Ethicon, Inc., Raritan, USA). The purpose of this study was to determine if 2-OCTA impedes bony healing. Osteochondral plugs in 38 retired Sprague-Dawley rats were created in both hind legs. Each rat had one plug dipped in 2-OCTA before fixation and one control plug. H&E staining was used to quantify bone bridging. The 2-OCTA group had a mean bridging bone circumference of 22.80%, significantly less than 67.75% in the control group (p<0.05). Our data suggests that 2-OCTA blocks bridging bone formation, making it a poor choice for fracture fixation.

## Introduction

Intraarticular fractures pose a significant challenge for the orthopedic surgeon. The articular cartilage is attached to a small piece of subchondral bone, making stable fixation of these fragments difficult. When the nonarticular bony fragment is too small to support stable fixation, other options must be explored. A number of techniques have been described, including headless screws [1], bioabsorbable darts [2], suture bridges [3], and transchondral screw placement [4]. All of these techniques have associated drawbacks in a variety of scenarios. Cyanoacrylate adhesives have been used in the past for the fixation of osteochondral fragments. Yilmaz and Kuyurtar reported using n-butyl-2-cyanoacrylate (Histoacryl®, B. Braun AG, Melsungen, Germany) for fixation of a talar osteochondral lesion with a good clinical result and MRI evidence of incorporation of the fragment at three months [5]. Kang et al. described using 2-octyl-cyanoacrylate (Dermabond®, Ethicon, Inc., Raritan, USA) for the fixation of small facial bone fractures with maintained alignment and no bone resorption at 13 months follow up [6].

Cyanoacrylate glue is an attractive alternative for the fixation of osteochondral fragments as it does not violate the cartilage surface, requires less technical skill, and is relatively less dependent on the depth of subchondral bone attached to the chondral fragment. Conversely, there are concerns about cytotoxicity from cyanoacrylate breakdown products [7] and the blocking of osteoprogenitor cells at the fracture interface [8]. This osteoprogenitor cell blockade could potentially lead to inferior bony healing due to the physical barrier introduced by the cyanoacrylate glue. It is not clear if this physical blockage will occur or if cyanoacrylate glue will resorb quickly enough as to not hinder bony healing. The purpose of this study was to determine if 2-octyl-cyanoacrylate (2-OCTA) resorbs from the fracture site in a reliable time-dependent manner and to provide insight if it can be used to stabilize articular fracture fragments without impeding bony healing based on bone bridging. We hypothesized that 2-OCTA would stabilize an osteochondral fracture fragment and allow for superior bony healing of the osteochondral fracture site compared to a control group in which no formal stabilization was employed, as measured by bridging bone formation at the fracture site. Additionally, we hypothesized that 2-OCTA would resorb from the fracture site in a time-dependent manner.

## Materials and methods

Thirty-eight retired Sprague-Dawley breeder rats were used for this experiment. Our research protocol was approved by our institution’s Institutional Animal Care and Use Committee (IACUC protocol 16-253.0-A). Rats were randomly divided into three-, seven-, and 28-day periods, with 10 three-day, 16 seven-day, and 12 28-day animals. Both hind legs were studied in all rats, with one leg serving as the control and the other as the 2-OCTA group in an alternating fashion. 

After allowing the animals to acclimate to their surroundings, they were taken to the operative suite and anesthetized with inhaled isoflurane anesthetic. The anterior knees were shaved and sterilized with betadine and 70% ethanol. We injected 1% lidocaine without epinephrine at the surgical site for local anesthesia, and cefazolin and buprenorphine were injected subdermally for antibiotic prophylaxis and postoperative analgesia, respectively. After preparing the sterile field, the cut finger of a sterile glove was used as a thigh tourniquet. The knee joint was accessed via a midline anterior incision and medial parapatellar arthrotomy. With the knee flexed, we then identified a point at the inferior aspect of the center of the trochlear groove, just superior to the intercondylar notch. Using a rotary tool (Dremel®, Mt. Prospect, USA) with a 2mm diameter trephine, an osteochondral plug 3mm in depth was created and removed. After ensuring meticulous hemostasis, the control plugs were reinserted. On the intervention side, the plug was grasped with forceps and dipped into 2-OCTA (Dermabond®) glue to cover the bottom and sides before being replaced into the dry donor site. The volume of 2-OCTA was the minimum to coat the bone-bone interfaces, with any excess being wiped away with gauze. Pressure was held until the unused glue was completely dry. On both knees, the arthrotomy was tightly closed using braided absorbable suture (Vicryl®, Ethicon, Raritan, USA) and the skin with permanent monofilament suture (Prolene®, Ethicon, Raritan, USA). Radiographs were obtained before the animals awakened. Postoperative analgesia was provided with buprenorphine (0.03mg/kg every 12 hours for 48 hours) injections and acetaminophen (250mg/kg for seven days postoperative) in the drinking water. No attempt was made to limit the rats’ activity or immobilize the involved extremities.

The animals were then euthanized at three, seven, or 28 days, as previously described. The femur was dissected free of any surrounding soft tissues. Repeat anteroposterior and lateral radiographs were obtained. High resolution photographs of the bone plugs were taken. The bone plug was gently probed and any gross motion was noted. The specimens were then fixed in 10% neutral-buffered formalin, decalcified in Immunocal (Statlab, McKinney, USA), and mounted in paraffin cassettes. Sections were cut axially through the plug in the distal femur, and two sections through the bone plug spaced 1mm apart were selected for analysis. The sections were then subjected to oil red O and hematoxylin and eosin staining protocols. For all analyses, p<0.05 was used to evaluate statistical significance.

**Figure 1:**
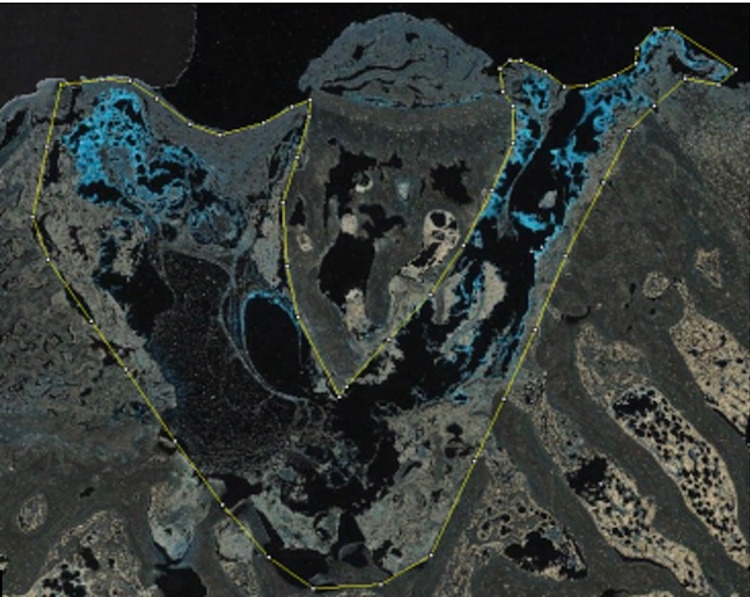
The area outlined in yellow represents the bone plug-femur interface, with the bone plug sitting at the center of the outlined interface. The articular surface is at the top of the slide. The blue stained material is cyanoacrylate, representing the presence of 2-OCTA. 2-OCTA: 2-octyl-cyanoacrylate

High-resolution images of all slides were obtained for digital analysis using the Aperio ScanScope XT slide scanning system (Leica Biosystems Imaging, Buffalo Grove, USA). Using ImageJ software (NIH, Bethesda, USA) the oil red O slides were analyzed for residual 2-OCTA (Dermabond®), as oil red O has been shown previously to stain cyanoacrylate red [9]. A polygonal area was drawn encompassing the bone plug-native bone interface. This area was then measured for percentage of red pigment as a percentage of total area (Figure 1). This was repeated three times for all sections and the results were averaged to obtain a percentage of residual 2-OCTA (Dermabond®) at the interface area.

**Figure 2:**
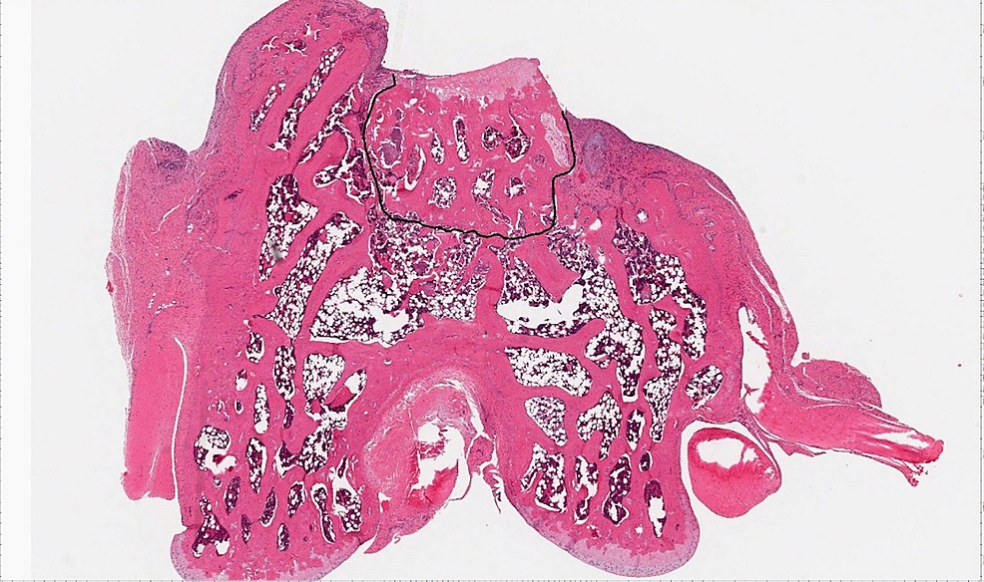
Measuring the bone plug circumference.

**Figure 3:**
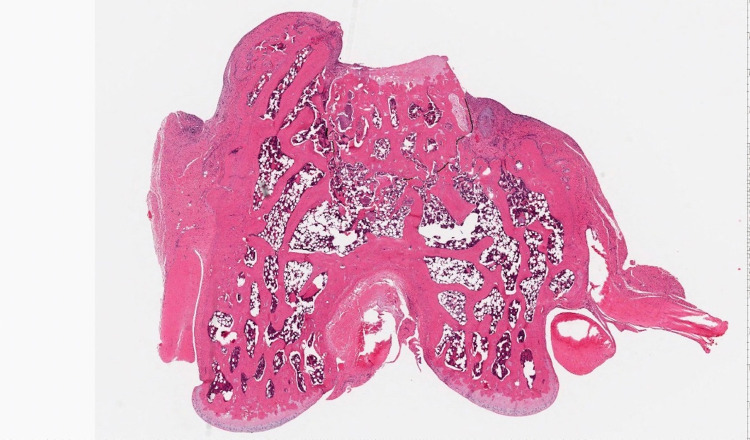
Measuring the length of bone bridging.

The H&E stained images were evaluated for presence of bridging bone using Imagescope viewing software (Leica Biosystems Imaging, Buffalo Grove, USA) Using the “Pen Tool,” the circumference of the bone plug was measured. If the articular surface of the bone plug was visible on the slide, it was not included in the measurement as no bridging bone would be expected there. The lengths of bridging bone circumferentially at the bone plug were summed and divided by the circumference of the plug to obtain a percentage measurement of bridging bone length (Figure 2, 3).

The fixation status of plugs as loose or fixed was analyzed by the Fisher Exact test at each time point and in aggregate. The Mann-Whitney U test was used to evaluate statistical significance of the bridging bone measurement, and a single-factor ANOVA was used to evaluate the difference between the three-day and the seven-day oil red O tests. 

## Results

There was no evidence of infection, wound dehiscence, or gross necrosis in the control or 2-OCTA groups. Grossly loose bone plugs are displayed by time period in Table 1. No group differences in the fixation status reached statistical significance independently at each time point or in aggregate across all time points.

**Table 1:** Loose and fixed implants with Fisher Exact test values. 2-OCTA: 2-octyl-cyanoacrylate

3-day					28-day			
	Fixed	Loose	Fisher			Fixed	Loose	Fisher
2-OCTA	10	0	1		2-OCTA	8	4	0.64
Control	9	1			Control	10	2	
7-day					Total			
	Fixed	Loose	Fisher			Fixed	Loose	Fisher
2-OCTA	8	2	1		2-OCTA	26	6	0.73
Control	9	1			Control	28	4	

Oil red O analysis

The mean percent area that stained red for cyanoacrylate was 45.7% in the three-day sections, versus 43.9% in the seven-day sections. When tested with single-factor ANOVA, the p-value was 0.74, indicating that the 2-OCTA volume had not decreased significantly between the three-day and seven-day groups.

Hematoxylin and eosin analysis 

Three samples (7L, 7R, and 8L) were excluded from analysis as the bone plug was not adequately visible at the cut depth of the slides. An additional sample (12L) was excluded as the bone plug had fallen completely out of the femur at the time of sacrifice. These excluded samples represented one sample from the 2-OCTA group and three samples from the control group. All other samples had at least one image of sufficient quality to be included in the analysis. The 2-OCTA group had a mean bridging bone circumference of 22.80% at 28 days, which was significantly less than the 67.75% in the control group (p<0.05). Similarly, the loose bone plugs had a mean bridging bone circumference of 12.9%, which was significantly less than the 53.07% of bridging bone formation in the fixed group (p<0.05).

## Discussion

The key finding of the study was the significantly diminished formation of bridging bone in the 2-OCTA group of 28-day rats. The rats in this group formed only a third of the bridging bone at the bone plug that was formed in the control group. There was also a trend towards more loose bone plugs in the 2-OCTA group, although this did not reach statistical significance. This would seem to indicate that the 2-OCTA interferes with bony healing, either through directly blocking osteoprogenitor cells or toxic metabolite formation. The use of the contralateral leg as a control makes these findings especially evident, such as in rat #13, where over 70% more bridging bone was present in the control leg than the 2-OCTA leg. 

Our evaluation of 2-OCTA resorption at the fracture interface indicates that minimal significant resorption occurs between the three- and seven-day time periods. On average, almost 50% of the interface area was blocked by cyanoacrylate during this time. This likely creates a barricade to the function of critical responses to bone healing on the cellular level, as cells would have difficulty migrating from the vascularized fracture bed to the dysvascular bone plug. Unfortunately, we do not have oil red O data available in the 28-day group. However, given the reduction in bridging bone seen in the 2-OCTA group at 28 days, it appears that this effect persists.

These findings are consistent with previous work by Esteves et al. in rat calvaria, which found decreased bridging bone formation and increased graft resorption at 60 days in the 2-OCTA group [8]. They postulate that 2-OCTA caused an increased inflammatory reaction, and the permanence of the adhesive in the interface did not permit neovascularization of the graft. This contrasts with the work by Shermak et al., which studied the effect of butyl-2-cyanoacrylate on the healing of rabbit parietal bone osteotomies [10]. They noted equal bone density in the cyanoacrylate group to those fixed with plates and screws and posited that the glue seemed to provide a scaffold for new bone formation as it degenerated.

There are a number of limitations to our study. No rats were followed past 28 days and it is possible that more of the grafts would have united at a longer time point as the 2-OCTA degraded. Our model provided relative stability to the bone plugs through the arthrotomy closure and extensor mechanism and a less stable model might have better highlighted the benefits of cyanoacrylate fixation. We were not able to directly evaluate the viability of the articular cartilage in our specimens, although viable articular cartilage is useless in the absence of a healed fracture fragment. A variety of other cyanoacrylate formulations are available for use and may have increased viability for this application; however, we chose to focus on 2-OCTA because of its wide availability and success with soft tissue closure.

## Conclusions

Overall, our data did not support our hypothesis that 2-OCTA would stabilize articular fracture fragments and allow for superior bony healing compared to an untreated control group. Our data suggests that the 2-OCTA does not fully resorb in the fracture site and thus blocks bridging bone formation in a 28-day period, suggesting it is a poor choice for fracture fixation. Though 2-octyl-cyanoacrylate is readily available clinically and presents an attractive option for fixation of small fracture fragments and articular cartilage with minimal subchondral bone, other fixation options may be superior.
